# Hemostatic Modification of the Bentall Procedure Using an Overlap Suture Technique

**DOI:** 10.1055/s-0039-1700998

**Published:** 2020-06-29

**Authors:** Mio Kasai, Akihiro Yoshitake, Hideyuki Shimizu

**Affiliations:** 1 Department of Cardiovascular Surgery, Keio University School of Medicine, Shinjuku, Tokyo, Japan

**Keywords:** hemostatic modifications, Bentall, overlap suture technique

## Abstract

Bleeding from the proximal suture line after replacement of the aortic root can be very difficult to control. Such bleeding is an important predictor of morbidity and mortality. Here, we detail a reliable method that is simple to perform and effectively prevents surgical bleeding. We adopted a technique of overlapping the pledgeted interrupted U-stitches of the proximal sutures on the prosthetic graft, which can eliminate the bleeding from the proximal suture line.

## Introduction

The description of aortic root replacement using a composite graft by Bentall and De Bono 1 enabled surgeons to offer a safer and more effective therapy for diseases of the aortic root. Subsequent modifications to this technique have substantially improved both short-term outcomes and long-term survival. However, bleeding from suture lines necessitating reexploration and multiple-unit blood transfusions remains a common cause of morbidity associated with the procedure, 2 3 especially when prosthetic valved conduits are used. We describe a modification to the Bentall technique that allows bleeding anastomoses to be identified and repaired before completion of the distal aortic anastomosis and release of the crossclamp. The “overlap suture technique” and “imbricated suture technique” have been previously described 4 5 ; however, our technique provides two important and novel improvements. The composite graft is created with a short skirt and the sutures are overlapped, not on the aortic annulus but only on the graft. We describe this as a reliable method that is simple to perform and effectively prevents surgical bleeding.

## Technique

All surgical approaches were conducted through a median sternotomy on cardiopulmonary bypass (CPB) via right atrial drainage and cannulation of the femoral or right axillary artery for arterial return. We instituted moderate hypothermia and infused cold cardioplegic solution from either the antegrade (directly into the coronary ostia) or retrograde (into the coronary sinus) direction, or both.

After reaching cardioplegic arrest, the aortic root aneurysm is excised, leaving only the two coronary buttons and approximately 5 mm of aortic wall attached to the aortic annulus. The abnormal aortic valve is also excised and the appropriate sized graft and valve selected.

Assistants make the composite graft in the operating room. The composite graft must have a short skirt that is 3 to 4 mm in length for sewing the proximal margin. After sewing the valve to the graft, fibrin-sealant spray is administered to the outside of the suture line of the composite graft.

Interrupted 2–0 polyester pledgeted U-stitches are then started on the annulus in an everting mattress fashion, with the pledgets lying on the outside of the annulus. Overlapping sutures are never made directly on the annulus to avoid the needle piercing the previous suture. Piercing the previous suture induces shear force on the annulus which may result in dissection of the annulus. All interrupted mattress sutures on the annulus are inserted in this fashion.

**Fig. 1:**
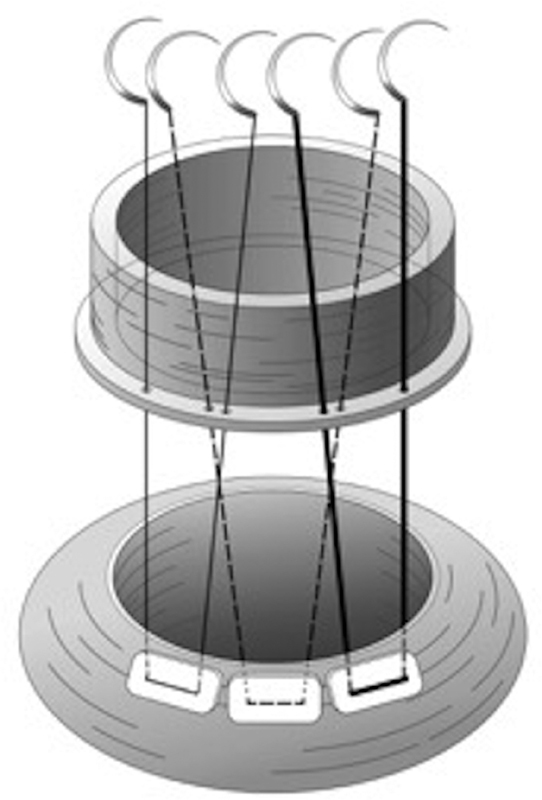
The overlapping mattress sutures are made on the composite graft but not on the aortic annulus. The first three mattress sutures are depicted as a thin line (first), a dashed line (second), and a bold line for clarity.

The composite graft is then brought to the operative field and the annular sutures passed through the sewing skirt of the composite graft. An overlap technique is used on the graft, placing the first needle of the subsequent mattress suture behind the second thread of the previous suture in the graft, overlapping by approximately 1 to 2 mm and using slightly larger bites than usual ( Fig. 1 ). Care must be exercised to avoid piercing the previous suture while overlapping.

When all sutures have been passed through the graft, the composite graft is lowered into the annulus and all sutures were tied and divided.

The proximal suture line can then be tested for bleeding by filling the left ventricular cavity with blood using the vent catheter and manually squeezing the left ventricle gently into the clamped aortic graft.

After confirming a watertight suture line, the left and right coronary buttons can be attached to the graft in that order. The left coronary button is attached to the graft by means of continuous 5–0 polypropylene sutures without Teflon felt pledgets. We then test the left coronary button suture line for bleeding by administering antegrade blood cardioplegia through the aortic graft.

After confirming hemostasis of the left coronary ostium, we perform the distal anastomosis, followed by suturing of the right coronary button to the aortic graft. The distal anastomosis and the right coronary anastomosis are performed with a running 4–0 polypropylene suture and 5–0 polypropylene suture, respectively. Before removing the aortic clamp, we spray fibrin sealant onto all suture sites.

After rewarming and deairing, the patients are weaned from CPB.

From February 2003 to March 2015, we performed the Bentall procedure using this technique on 71 consecutive patients, 13 (18.3%) of whom were emergent cases (aneurysm rupture, Type A dissection), and 30 (42.3%) of whom required aortic arch replacement. None of these patients needed surgical reexploration. In-hospital death occurred in only one patient due to acute coronary syndrome.

## Comment

Bleeding at the level of the proximal anastomosis remains a problem with the Bentall procedure, especially when the bleeding originates from the posterior aortic circumference. That portion of the aorta is almost impossible to reach after completion of the procedure. Mobilizing the graft after left main coronary artery anastomosis is a high-risk maneuver.

Recently, many modifications of the original technique have been proposed to prevent the problem. The “overlap suture technique” and “imbricated suture technique” have already been described in previous reports. 3 4

**Fig. 2:**
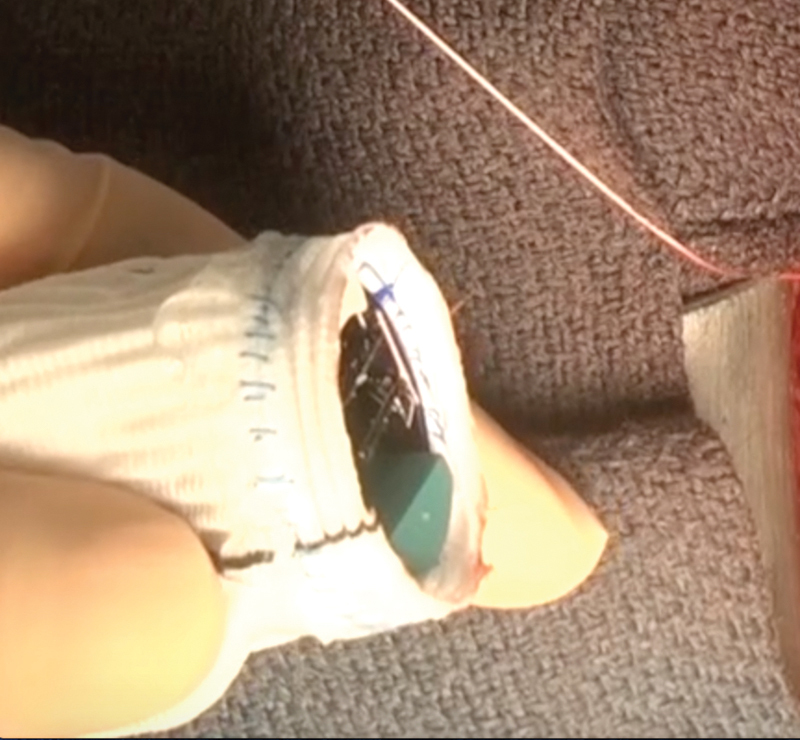
A composite graft (Dacron graft with sutured mechanical valve) with a short skirt.

**Fig. 3:**
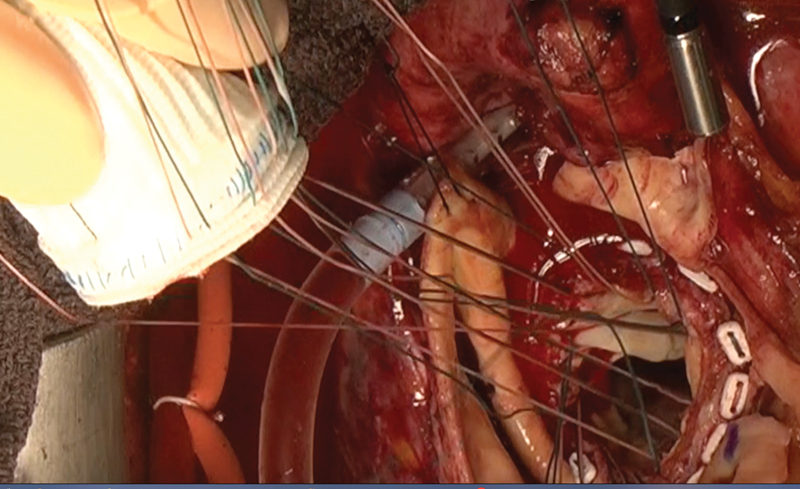
Overlapping mattress sutures are used on the composite graft but not on the aortic annulus.

However, our technique differs from these previous descriptions. Our technique has two important distinctions as follows: (1) the composite graft is created with a short skirt (3–4 mm) and (2) the sutures overlap on the graft but not on the aortic annulus ( Figs. 2 , 3 ). The sutures are overlapped only on the graft due to shear forces potentially created by overlapping sutures on the annulus which may induce the dissection of the annulus if the sutures are tied too tightly. The prosthetic grafts have sufficient tolerance to the overlapping shear forces. Compared with the previously proposed hemostatic modifications of the proximal anastomosis, 6 7 8 our technique does not call for the use of additional material or adjunctive sutures, which can lengthen crossclamp and CPB times, potentially canceling out the effect of the technical modification itself by impairing coagulation and promoting postoperative morbidity.

In conclusion, our surgical technique is simple to perform and provides an effective means to prevent surgical bleeding.

**Editor's Questions**

1. *Do you feel that adding the 3-mm skirt is important? Why is it not sufficient to pattern your sutures in the same way, but place them into the valve cuff of a conventional mechanical valved conduit?*
  It is important because the valve cuff is so thick that overlapped suture might pierce the previous suture in the valve cuff. On the other hand, graft skirt is thin enough to make it easy to confirm the suture is not piercing the previous suture while overlapping.
2. *Do you use the same method for biological valves, as well as mechanical valves?*
  Exactly, we use the same method for biological valves, as well as mechanical valves.

## Notes

Editor's Comments M.K. focuses on a very real problem in performance of the Bentall procedure. Bleeding from the proximal suture line on the annulus can occur, and, when it does, it can be catastrophic. Methods to discourage such bleeding are welcome.

## Notes

Editor's Questions

1. *Do you feel that adding the 3-mm skirt is important? Why is it not sufficient to pattern your sutures in the same way, but place them into the valve cuff of a conventional mechanical valved conduit?*
  It is important because the valve cuff is so thick that overlapped suture might pierce the previous suture in the valve cuff. On the other hand, graft skirt is thin enough to make it easy to confirm the suture is not piercing the previous suture while overlapping.
2. *Do you use the same method for biological valves, as well as mechanical valves?*
  Exactly, we use the same method for biological valves, as well as mechanical valves.
